# Macronutrient distribution in soil subjected to raw sanitary sewage application by closed-end furrows

**DOI:** 10.1038/s41598-023-38528-5

**Published:** 2023-07-13

**Authors:** Marcus Vinícius Araújo Marques, Thiago Henrique Ribeiro Silvério, Ana Paula Miranda Pereira, Marcos von Sperling, Thiago de Alencar Neves

**Affiliations:** Department of Sanitary and Environmental Engineering, Federal University of Minas Gerais (Universidade Federal de Minas Gerais), Belo Horizonte, Minas Gerais Brazil

**Keywords:** Environmental monitoring, Pollution remediation, Geochemistry

## Abstract

This work aimed to evsaluate the influence of raw sanitary sewage (RS) application in closed-end and level-bottom furrows on the distribution of macronutrients and sodium in the soil along its depth. The applied amount of RS was controlled due to the harmful effect of sodium, with a weekly application over three consecutive years. The experimental design consisted of two experimental plots receiving RS, one with alternating the site of effluent application (alternation of inlet and outlet—TFA) and the other without alternating the site of application (only inlet—TFN); and two experimental plots receiving conventional mineral fertilization, both irrigated with public water supply, where one alternated the site of water application (alternation of inlet and outlet—TWA) and the other without alternating the site of application (only inlet—TWN). The inversion of the flow direction with RS application in the closed-end furrows (TFA) provided a better distribution of nutrients in the soil along its length. There was no significant difference in the levels of macronutrients and sodium between the initial and final regions of the furrow soil. In contrast, the fixed flow direction experimental unit (TFN) exhibit a significant difference between the initial and final levels of total nitrogen, phosphorus, potassium and sodium in the closed furrows with mean concentrations of 265.2, 16.2, 46.7 and 110.0 mg dm^−3^, respectively.

## Introduction

Demand for water and mineral fertilizers for agricultural production has increased significantly in recent years due to uncontrolled population growth and climate change. The reuse of water for agriculture or for soil fertilization is a reality and there are still several barriers related to the quality of the water used and the environmental impacts resulting from these applications, such as the contamination of groundwater by infiltration processes and of surface water by runoff into the soil^1^.

Problems related to water scarcity and high demand for mineral fertilizers have been discussed by researchers around the world. As an alternative solution, the application of raw sewage can be considered, supplying the nutritional needs of plants, in addition to supplying part of the water needs of plants, and taking into account that sewage is available throughout the year in cities that have a sewerage system^2–4^.

When it comes to the application of raw sewage in agriculture, the conventional methods of localized application (sprinkling and dripping) can present clogging problems due to the blockage of the system by chemical, physical and biological agents, resulting in the requirement of a treatment step for potential uses. Also, the need for reduction of solids and increase of flows for better performance of these techniques in the application of raw effluents will increase the workforce, make their use more expensive or unfeasible^5,6^. Therefore, the use of furrows for the application of raw sewage can be considered adequate because it presents a low risk of obstruction combined with sanitary safety as there is no contact between the plant and the sewage^7–10^.

The problem of furrow fertigation is linked to the way in which nutrients are distributed in the soil, as they cause a greater opportunity time in the entry region. Therefore, the initial region of the furrows will present a higher concentration of nutrients and a greater risk of leaching. This can be confirmed with studies that point out this difficulty due to the accumulation of nutrients at the entrance of the furrows^11,12^. However, when working with closed-end furrows, this tendency may change, with greater accumulation of nutrients in soil in this region^13^. Many researchers have dedicated their time to optimizing the furrow application system, which is the subject of this work^13–22^.

At the time of application of RS in the soil, the macronutrients present in its composition are highlighted because they are in greater quantity, where the safety of the application process lies in the low accumulation of these nutrients in the soil, guaranteed by the extraction capacity of plants involved in the process. It should be noted that the use of raw sewage in the irrigation of agricultural crops without prior treatment is a prohibited practice in several countries, however some countries allow this practice. Studies have shown that the use of wastewater without due care (supplying the water needs of the plants with the application of untreated wastewater) can cause accumulation of toxic metals, leaching of pollutants and intoxication of plants^4,6,23–25^.

Studies have demonstrated the risks of nitrogen (N) leaching into the soil after the application of sewage, evaluating the losses and their accumulation as a function of the applied dose and the local water regime (irrigation depth and precipitation), in addition to the soil characteristics^13,19,20,22,26–28^. On the other hand, studies indicate that phosphorus (P) in the soil that receives sewage has low soil leaching, due to high interactions with the environment^29,30^. Therefore, studies that bring solutions to reduce nutrient leaching in the soil have been topics of discussion.

Traditionally, the application of wastewater by furrows has as its principle the generation of only one flow direction, originating at the point of highest elevation following towards the lowest elevation. The innovation of this work lies in studying the change of flow direction in the furrows at each application (weekly), as these were built with closed ends and leveled. This system modification was studied with the application of raw sewage (untreated) for a long period (3 years), which has not been found in the literature so far.

The objective of the present work was to evaluate the influence of RS application in flat bottom furrows with closed ends (alternating flow inlets and outlets) on the macronutrient content along the depth in the soil in comparison with the results obtained with conventional mineral fertilisation (CMF), during 3 years of uninterrupted experimentation.

## Materials and methods

The experiment was conducted in an area at the Wastewater Treatment Plant of the Minas Gerais Sanitation Company (*Companhia de Saneamento de Minas Gerais—COPASA ETE—Onça*), located near “Ribeirão do Onça” river, in the metropolitan region of Belo Horizonte, Brazil. The geographic coordinates of study are 19°49′20.6″ South and 43°53′46.6″ West, at an elevation of 852 m.

The study region has a humid tropical climate, with most rainfall during the summer. The average annual air temperature (T_mean_) was 23ºC, average annual relative humidity (RH_mean_) was 61%, and average annual accumulated precipitation (Pr) was 1353 mm during the experimental period^31^. The soil under study according to the Soil Taxonomy of the United States Department of Agriculture, has characteristics similar to an Inceptisol^32^.

**Figure 1:**
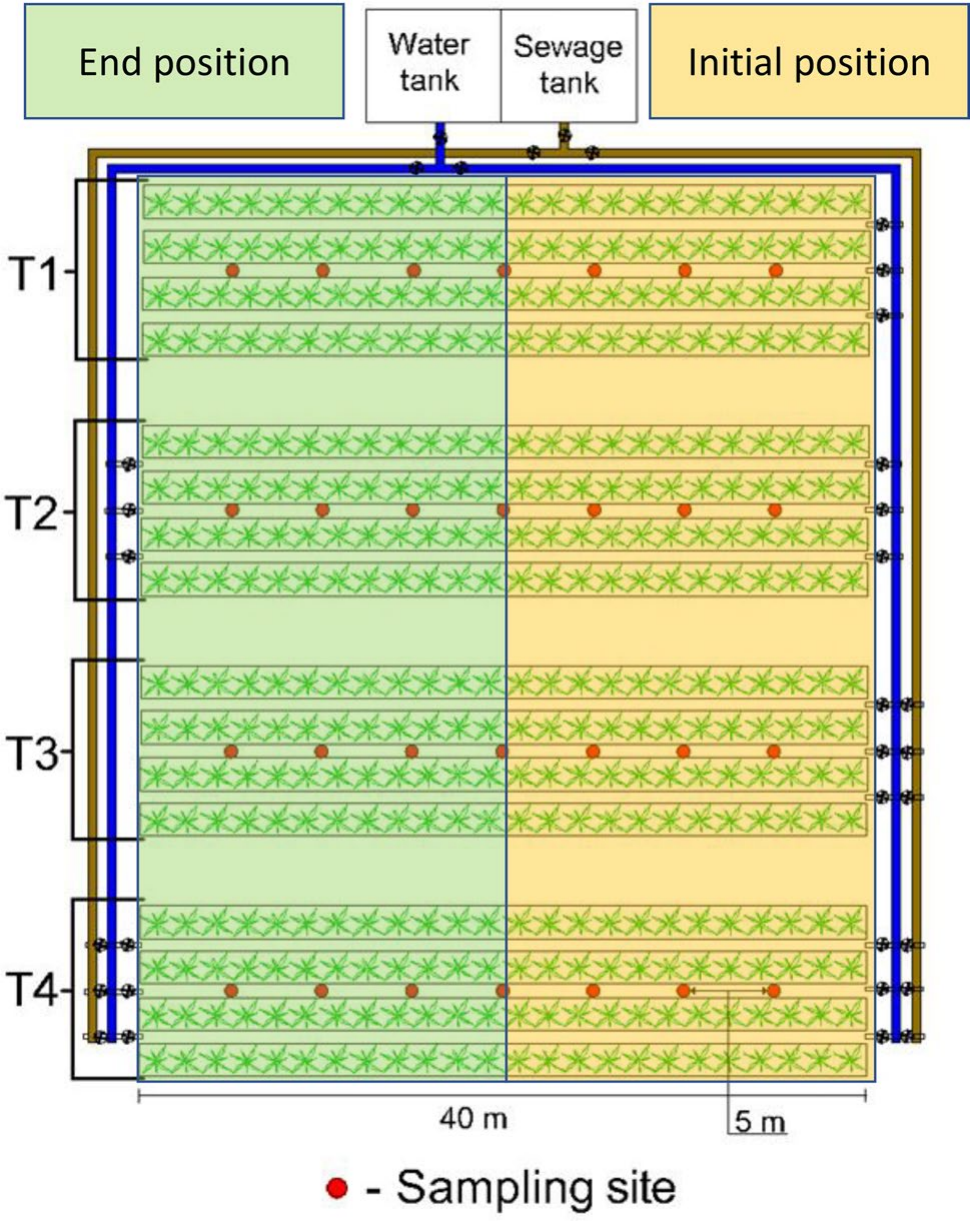
Schematic of the experimental area with indication of the sites where the soil was sampled.

The use of plants in the present study complies with international, national and institutional guidelines. Elephant grass (*Pennisetum purpureum*) was planted in June 2016, from plant stems. The experiment design consisted of four treatments and seven sampling regions along the length of the furrows. The treatments considered were: (T1) conventional mineral fertilization (CMF) associated with treated water irrigation (TW) at fixed flow direction in the furrows (TWN); (T2) CMF and TW irrigation with alternating flow direction in the furrows (TWA); (T3) Fertigation with raw sewage (RS) associated with TW irrigation, both applied at fixed flow direction in the furrows (TFN); and (T4) Fertigation with RS associated with TW irrigation, both applied with alternating flow directions in the furrows (TFA). Each experimental plot was 72 m^2^ (total of 288 m^2^), which consisted of three furrows and four planted rows. The lateral furrows and lateral planted rows were disregarded in the analyses since they were used only as “borders” to reduce external interference with the system under investigation. The length of the furrows was 40 m, with a spacing of 0.6 m between them, built on ground level and with closed ends (Fig. 1).

CMF treatments received doses of 70 kg ha^−1^ year^−1^ of P applied manually at the beginning of each year, and 300 kg ha^−1^ year^−1^ of N and potassium (K) also applied manually, after cutting the aerial part of plants. The CMF was acquired locally and is based on simple superphosphate (Ca(H_2_PO_4_)_2_ + CaSO_4_.2H_2_O), urea and potassium chloride, in accordance with the recommendations of the Soil Fertility Commission of the State of Minas Gerais^33^.

To control salinity, fertigation with RS was performed weekly by applying a sodium dosage equivalent to 300 kg ha^−1^ year^−1^ of Na^34^. Irrigation and complementing water application to the plots fertigated with RS were also performed weekly, and evapotranspiration was used to calculate the water demands of the plants^35^.

It should be noted that the central proposal of this work was to evaluate a new form of management for furrow fertigation in the application of raw wastewater (TFA treatment), where the operation consisted of applying RS alternating the flow direction between the beginning and end of the furrows. The change of application position of TW or RS that corresponded to the change of application between the two ends of the furrows was possible due to the construction of flush furrows. It was decided to apply RS associated with TW in the fertigated treatments, in which the non-erosive maximum flow rates and flow rates established for this type of system were respected. This method of operation ensured that the RS infiltrated quickly and was only briefly exposed to the soil surface.

The experiment was conducted for a period of 3 years, from June 2016 to July 2019, during which periodic cuts of the aerial part of the plants were made whenever the plants of all experimental plots reached a height greater than 1 m. In addition, soil collection was performed at seven different positions along the length of each furrow, at depths of 0–0.2, 0.2–0.4 and 0.4–0.6 m, to obtain results related to the leaching of macronutrients in the soil profile.

The RS was collected weekly and before its application in the furrows, sodium (Na), total Kjeldahl nitrogen (TKN), total phosphorus (TP) and potassium (K) concentrations were determined (Table 1). All laboratory analyses were performed according to the methodologies proposed by APHA^36^.

**Table 1:** Mean concentration of sodium (Na) and macronutrients (N, P and K) in raw sewage, in addition to the amounts applied via fertigation and conventional mineral fertilization during 3-years of experimental period.

Parameters	Average concentration (mg L^−1^)*	Fertigation	Mineral fertilization
		Input (kg ha^−1^ year^−1^)	
Na	71.4 (± 21)	300	–
N	148.9 (± 33)	647	300
P	17.8 (± 6)	86	70
K	38.5 (± 11)	176	300

* n = 130; values in parenthesis are the standard deviations.

The chemical and physicochemical characteristics of the studied soil were the available levels of Na, P and K, the total levels of TN and organic matter (OM) and the values of electric conductivity (EC) and hydrogenionic potential (pH). All laboratory analyses were performed according to the methodologies proposed by EMBRAPA^37^. The initial characterization of the soil in the studied area showed average pH levels of 7.2, EC of 47 µS cm^−1^, OM of 1.2 dag kg^−1^, TN of 201.7 mg kg^−1^, P of 4.1 mg dm^−3^ and K of 61.3 mg dm^−3^.

Statistical analyses of the studied soil were carried out in the three consecutive years after the system was implemented (2017–2019). These data were submitted to tests of central tendency, using analysis of variance (ANOVA), comparing the initial and final region of the furrows, and the depth of the soil, using Student's t-test for paired comparison, and Tukey's test for multiple comparisons, both with 5% significance level. The statistical analyses and graphs presented in this work were generated using the STATISTICA software, version 7.

## Results and discussion

### Chemical characteristics of the surface layer of the soil

**Figure 2:**
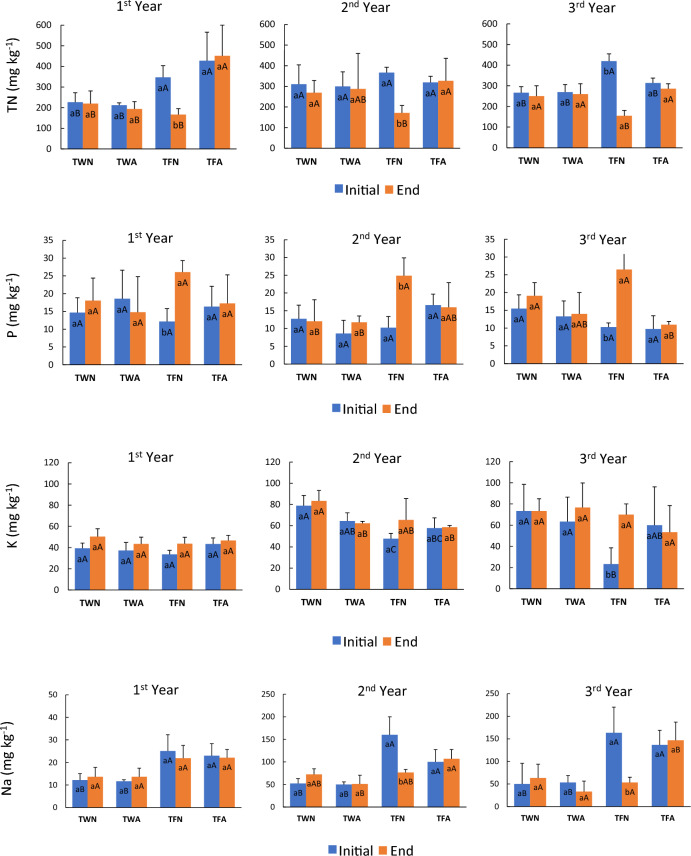
Total nitrogen (TN), available phosphorus (P), available potassium (K) and available sodium (Na) content in the surface layer (0–0.2 m) of the soil, considering the initial and end regions of the furrows submitted to fertigation with raw sewage, for a period of 3 years of experimentation. Comparison between the initial and final region of the furrows, where each treatment with the same lowercase letter did not present a significant difference, by Student's t test (α = 0.05; n = 9). Comparison by region, initial and final, of the furrows between treatments with the same capital letter did not show a significant difference, by the Tukey test (α = 0.05; n = 9). Error bar shows the standard deviation of the samples. TWN—Water irrigation treatment, without alternating the application position in the furrows; TWA—Irrigation treatment with water, with alternation in the position of application in the furrows; TFN—Fertigation treatment with RS, without alternating the application position in the furrows; TFA—Fertigation treatment with RS, alternating the application position in the furrows.

Observing the data presented in Fig. 2, the TFN treatment, in which there was no alternation in the flow direction of RS application in the furrows, showed a significant difference of the TN content in the surface layer (0–0.2 m), considering the initial and end of the furrows during the entire sampling period.

The strategies adopted in this experiment were based on the amount of RS applied in the TFN and TFA and were stipulated as a function of the Sodium (Na) dose, which was 300 kg ha^−1^ year^−1^, which generated input doses of 647, 86 and 176 kg ha^−1^ year^−1^ of N, P and K, respectively (Table 1). The water needs of these treatments were complemented with treated water, and, as the furrows were built with closed-ends and leveled, an accumulation of water was noted throughout the entire length of the studied area.

The average TN contents in the initial region of the TFN furrows between the 1st, 2nd and 3rd year of experimentation were 347 (± 56), 367 (± 26) and 420 (± 35) mg kg^−1^, respectively. This increase in TN content in this region shows that fertigation, when done in a conventional way, that is, always at the initial end of the furrows, causes nutritional enrichment in the soil of this region, increasing the risk of leaching. In the 3rd year, the TFN treatment showed a significantly greater difference, proving the accumulation of this element in this initial region of the treatment (Fig. 2).

In a study of the application of untreated municipal wastewater to supply the water demand of plants through furrows with a closed end and single flow direction (which unlike the TFN, has a downward slope), the researchers observed the accumulation of wastewater in the final part of the furrows (downstream) and, as a consequence, this region presented in the superficial layer (0–0.25 m) a higher total N content in the soil after corn cultivation^13^. It can be deduced that the act of supplying the water demand of plants with wastewater and building sloped furrows alters the profile of accumulation in the soil, which for the TFN was in the initial part of the furrows where the limitation of wastewater application was the Na dose with a complement of treated water.

There are several strategies to increase the efficiency of water distribution in furrows. The management and distribution of water in the furrows must consider the variation in the volume and flow applied, irrigation time, cutting off the discharge flow, before reaching the end, and the advance time of the application, as well as the constructive characteristics of the furrows, such as length and closed ends^21^. Therefore, the proposal to modify the TFA, alternating the flow direction in the furrows, increases the distribution of water and nutrients involved in the present fertigation.

The TFA showed a reduction in the distribution of TN contents in the surface layer of the soil at the beginning and end of the furrows, with a mean reduction from the 1st year to the 3rd year, ranging from 429 to 300 mg kg^−1^, respectively (Fig. 2). This reduction is indicative of a better distribution of this nutrient in the soil, which implies that the plant was able to extract this nutrient over the years when applied alternately in relation to the RS application flow direction.

In a study carried out with the application of treated sewage used in the fertigation of pumpkins, applying 150% of the plant's water demand, a reduction of approximately 30% was observed in the TN content of the surface layer of the soil^38^, similar to those obtained in the areas submitted to TFA, in this work. The authors associated this reduction to the productive stimulus of this wastewater applied locally, the same happening concerning TFA, which provided an increase in crop productivity due to a better distribution of nutrients in the soil.

The crop cultivated here in this work was elephant grass (*Pennisetum purpureum*) and the dose of macronutrients applied for its development was shown in Table 1. For the system to be safe in terms of nutrient accumulation and leaching, the cultivated plant must be capable to absorb the applied nutrients. A study carried out in the application of RS cultivating elephant grass showed that this species has the capacity to extract 688, 102, 508 kg ha^−1^ year^−1^ of N, P and K, respectively^39^. This justifies the low accumulation in the surface layer of the TFA and emphasizes the risk of leaching in the initial region of the TFN, which will be seen in the next topic.

Total nitrogen is present in the soil in the form of nitrate (electronegative), ammonium (electropositive) and organic N. Because of this, there is a tendency for researchers to study the leaching of the nitrate fraction because it is in greater relative concentration due to the oxidative profile of the soil, which in most systems has a high mobility and can pollute soil and groundwater^12,13,20,23,26,40^. Previous studies showed that TN contents can vary from 600 to 5600 mg kg^−1^ in the surface layer of cultivated soils^41,42^. The results in the present study indicate that TN contents are low and that the applied dose of CMF and RS could be considered ideal for the plants, without causing an excess of this nutrient in the soil. A study with fertigated soils with treated sewage in Jordan showed that these values can vary between 300 and 600 mg kg^−1^, corresponding to the findings in this work^43^.

The average TN levels presented in all treatments were below 600 mg kg^−1^, comparable to a value found in a study after a long period of application (20 years) of treated sewage in the soil^44^, but above 154 mg kg^−1^, a value found in another study with RS receiving soil for 1 year^45^. This variation is common in terms of soil TN because this nutrient has a high mobilization capacity and its accumulation in the soil profile will depend on the characteristics of the soil, the culture management adopted and the time of application represented by the applied load. Even so, the application from RS normally has shown higher accumulation in the entire profile, especially in the long-term^46^.

A study comparing fertigation with treated and untreated sewage showed the superiority of untreated sewage in increasing the percentage of OM and TN in the soil^47^. The results observed by these authors confirm the advantages of not treating domestic sewage for use in agriculture, always respecting the sanitary safety of the practice.

There is an environmental concern with soil N, since it can be leached, and this occurs with the excessive application of this fertigation route. One study showed that in a soil receiving treated sewage, the concentration of TN was increased by about 4.6 times, reaching levels of 12,000 mg kg^−1^ with 10 years of application, when doses were applied to meet the crop's water demand^48^.

The TFN provided a contrary trend of distribution in the soil between the contents of available P when compared with the contents of TN in the superficial layer of the soil, that is, the samples of the final region of the furrows showed higher levels of available P throughout the experimental period. These values are the result of the greater availability of TN in the initial region of the furrows, which stimulated a greater extraction of P by the plants, leaving the final region of the furrows with higher contents and with low variation in these contents, over the years (Fig. 2).

The initial region of the treatments showed no significant difference in P contents over the years of experimentation. However, the final region of the TFN treatment showed a significant difference in relation to the TFA in the 3rd year of experimentation, a result of the optimization of nutrient extraction by the TFA plants, combined with the low extraction efficiency in the TFN (Fig. 2).

The contents of P available in soil samples collected in the surface layer of treatments that received CMF (TWN and TWA) did not show significant differences when comparing the initial and final regions of the furrows (Fig. 2). Because of the way the fertilizer was applied, it was protected by the low mobility that P has in the soil, due to the adsorptive interaction with the colloids present in this system^49^.

The available average levels of P obtained in the plots that received RS in the 1st year of experimentation were approximately 15.0 mg dm^−3^, with similar values to a study that also applied RS in a soil in Jordan^50^. In a study conducted with the fertigation of domestic sewage treated by different methods, applied to supply 100% of the water demand of the pepper crop, the authors found available P levels in the range of 150 to 230 mg dm^−3^ of soil, demonstrating the inappropriateness in regarding the establishment of domestic sewage application ranges based on water demand and not on its reference chemical element^51^. Researchers fertigated crops and observed a significant increase in the available P content in the soil, which went from 18 to 41 mg dm^−3^ in 16 weeks of treated domestic sewage application^52^.

The available P is a good indicator of successive RS applications, as it is a low-mobility element in the system. In general, the proportions in which they are found in RS are relatively low for cultural needs, if compared to the other macronutrients, and for this reason RS applications of up to 30 years do not present P levels in the soil higher than 18 mg kg^−1^^46^. Therefore, it is believed that there is a low risk of P leaching in the TFA treatment along the entire furrow and a moderate risk of P leaching in the initial region of the TFN furrows.

There was an apparent accumulation of available K in the surface layer of the soil of the furrows, from the 1st to the 2nd year, but this trend was not maintained in the 3rd year of experimentation. All treatments did not provide significant differences in K contents, considering the initial and end of the furrows, with the exception of TFN, in the 3rd year, in which the K contents of the initial end of the furrow were significantly lower than those of the final end. This difference is related to the same reason discussed in relation to the P content, since the amount of N made available in this initial region stimulated the extraction of K, resulting in a deficit condition, which did not happen in relation to TFA.

The average contents of available K provided with RS application are lower than those obtained in a study in Jordan, which were 350 to 1,000 mg dm^−3^ in the fertigation of olive trees with RS^50^. A similar result was obtained in soil fertigated with treated sanitary sewage for 10 years, in which this value reached 681 mg dm^−3^^48^. In RS applications studying four types of soils from 10 to 30 years of application in Egypt, the average value was 374 mg kg^−1^^46^.

Comparing the treatments in the 3rd year of experimentation, the Na content in the initial region of the furrows was significantly higher in treatments that received RS (TFN and TFA), compared to treatments that received conventional mineral fertilisation (TWN and TWA). However, in the final region of the furrows, only the TFA treatment showed a significant superiority in the contents of this element (Fig. 2).

The contents of available Na obtained in soil samples collected in the area subjected to TFA increased over the years, with an average of 22, 106 and 143 mg dm^−3^, for the 1st, 2nd and 3rd year of experimentation, respectively. It is important to emphasize that this increase did not cause any apparent problem to the soil, given that productivity was maintained.

A study that applied treated domestic sewage to supply the water demand of sugarcane obtained available contents in the order of 108 mg dm^−3^ of Na in the soil, a value higher than the 1st year of application of RS, and exceeded in the following years, in soil samples collected at the beginning of the furrows in the TFN and, in the 3rd year of the samples collected from the areas submitted to the TFA^53^. The presence of Na in the surface layer of the soil subjected to TFA may be an indication that there was no deep leaching of this chemical element in this system.

**Figure 3:**
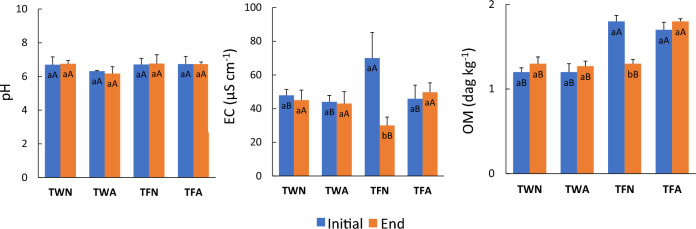
Hydrogenionic potential (pH), electrical conductivity (EC) and organic matter (OM) in the surface layer of the soil (0–0.2 m), considering the beginning and the end of the furrows submitted to fertigation with raw sewage, after 3 years of experimentation (reference year: 2019). Comparison between the initial and final region of the furrows, where each treatment with the same lowercase letter did not present a significant difference, by Student's t test (α = 0.05; n = 9). Comparison by region, initial and final, of the furrows between treatments with the same capital letter did not show a significant difference, by the Tukey test (α = 0.05; n = 9). Error bar shows the standard deviation of the samples. TWN—Water irrigation treatment, without alternating the application position in the furrows; TWA—Irrigation treatment with water, with alternation in the position of application in the furrows; TFN—Fertigation treatment with RS, without alternating the application position in the furrows; TFA—Fertigation treatment with RS, alternating the application position in the furrows.

Observing Fig. 3, the pH did not present a significant difference between the soil regions, including the treatments, which were close to neutrality (pH = 7). Several authors showed that the pH of soils receiving RS can change with application time, but, with longer application periods, it tends to approach neutrality, as observed in this experiment^29,50^.

EC indicates the amount of free ions present in the soil, which is expected to increase with the application of mineral fertilizers and RS. The results showed that the TFN treatment showed a significant difference between the initial and final region of the soil and, when compared to the other treatments, the initial region presented higher values, while the final region presented lower values (Fig. 3).

The increase in OM by the application of RS in fertigated systems has already been observed by several authors. Studies have observed that the soil OM started to be increased after 2 years of application of treated domestic sewage^54^, while other researchers observed faster increases, with considerable increases being observed with 6 months of application of raw domestic sewage^45^.

Only the TFN treatment showed a significant difference between the initial and final regions, a result of the poor distribution of RS caused by the form of application. The highest concentrations of OM were found in TFN in the initial region of the furrows, and in TFA in both regions, with values approaching 2 dag kg^−1^. A study carried out in Pakistan in areas that received RS for long periods showed that the value of OM was increased, being similar to that of this work^55^.

OM is responsible for improving the physical structures of the soil. The concentration of OM in the soil is responsible for the reduction of the availability of toxic metals (such as copper, zinc, cadmium and lead). Toxicity studies demonstrated that the application of RS in crops consumed raw increased their concentration in plant tissues^1,24,25^. Thus, the increase in OM observed in the TFA will increase security with regard to the reduction in the availability of heavy metals in this system.

### Chemical characteristics of the soil along its depth

**Figure 4:**
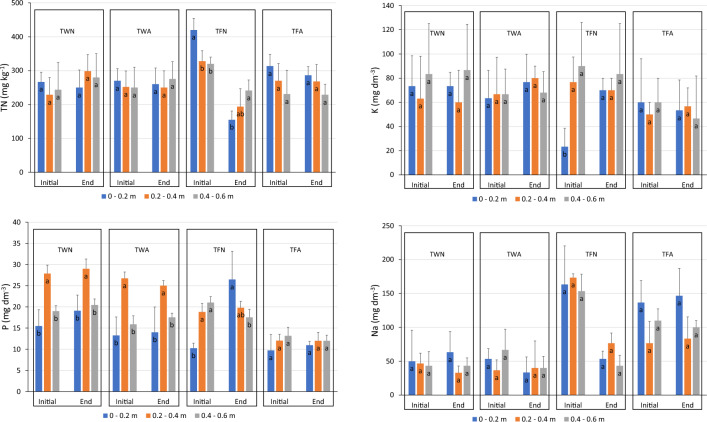
Total nitrogen (TN), available phosphorus (P), available potassium (K) and available sodium (Na) content at different soil depths (0–0.2, 0.2–0.4 and 0.4–0 0.6 m), considering the initial and end regions of the furrows subjected to fertigation with raw sanitary sewage, after 3 years of experimentation (reference year: 2019). Comparisons in soil depth in each region with the same letter do not show a significant difference, by the Tukey test (α = 0.05; n = 9). Error bar shows the standard deviation of the samples. TWN—Water irrigation treatment, without alternating the application position in the furrows; TWA—Irrigation treatment with water, with alternation in the position of application in the furrows; TFN—Fertigation treatment with RS, without alternating the application position in the furrows; TFA—Fertigation treatment with RS, alternating the application position in the furrows.

Intending to analyze the profile of accumulation along the soil depth of the elements under study, Fig. 4 presents the soil concentration of TN, P, K and Na within three different layers. The treatments that received conventional mineral fertilization showed no significant difference between the soil layers for both initial and end regions of the system.

In the final region of the TFN, the surface layer of the soil (0–0.2 m) presented low TN content, with a significant tendency to increase in depth, which can be explained by the nutritional demand of plants in this region having been greater than the contribution via RS (Fig. 4). This result demonstrates the difficulty of working with fertigation in a conventional way using RS, as the final region of the furrows tend to present low productivity due to nutritional deficit, as demonstrated in studies that presented forage productivity^39^.

TFA showed homogeneous results in TN levels along depth without significant differences (Fig. 4). This demonstrates a good distribution of this nutrient along the furrows, with the results indicating that there was no leaching of nitrogen in this treatment.

The effects of RS application on TN concentration along the soil profile were noticed by researchers that tested the application of this wastewater in different soils, resulting in its tendency to accumulate in the deeper layers due to its low interaction with the soil. However, the intensity of this interaction depends on the type of soil worked with^47^. Inadequate management has caused TN enrichment along the soil profile of areas that received treated domestic sewage in Jordan, reaching 558 mg kg^−1^, with values higher than those in this work^40^.

It should be noted that the shapes of the furrows may be involved in the leaching rates, as some studies indicate that a higher ridges tend to increase these rates^19^. Some techniques can be used to reduce the leaching rates, such as the use of soil cover (with plastic), the reduction of the applied water depths, the reduction of the fertilization dose, the splitting of the applied doses. However, one must be careful not to reduce productivity^20,56^.

Therefore, the improvement in the distribution capacity of macronutrients in the TFA without significant accumulation in depth relies in a good water distribution along the furrows, which stimulates a homogeneous evapotranspiration and reduces the leaching of more irrigated points, as well as the improvement of the extraction of nutrients by the plants involved in the process that received their weekly fertilizer doses.

Figure 4 shows a significant accumulation of P in the intermediate layer (0.2–0.4 m) in treatments that received conventional mineral fertilisation (CMF). This evidences that the phosphate fertilisation was greater than the demand required by the plants, and the continuous application of this fertilizer could lead to deep leaching, however at a low rate.

The high availability of TN in the surface layer of the soil (0–0.2 m) in the initial region of the system demanded proportionally larger amounts of P, reducing its levels in this region, as shown in Fig. 4. The P contents in the soil presented for the treatment with alternation in the position of application of RS (TFA) were homogeneous, with no significant difference in depth in both regions (initial and final). This result can be justified by the better distribution of macronutrients in the soil, optimizing P extraction, which resulted in a lower average content across the entire profile, around 10.3 mg dm^−3^.

A study in which sewage with different levels of treatment was applied to crops in Egypt showed the accumulation of P throughout the soil profile, since it was applied to meet the water demand of plants, not respecting the nutritional need^57^. However, the study points out that this accumulation of P was restricted to the first 30 cm of the soil^58^.

As can be seen in Fig. 4, which shows the standard deviations of K contents, there is a high variability along the depth in the system for this element. This result is associated with the high mobility of K in the system, due to the weak bonds that monovalent cations present with soil structures.

It can be seen in Fig. 4 that the contents of K along the depth in the TFN treatment without alternating the position of application of RS presented a behavior similar to the P content in the initial region of the system, justified by the already mentioned stimulus of the excess of TN. However, the final region of this treatment showed no significant difference, with an average across the entire soil profile of 74.4 mg dm^−3^.

The RS, because it is a Na-rich wastewater, modified the content of this element in the soil of the treatments that were fertigated, and showed an increase of this element in its profile, as can be seen in Fig. 4.

The lack of a significant difference in Na in the initial region along the depth indicates that there was leaching of this element, which was expected by the load delivered via RS in this region of the soil. The same accumulation of Na in the soil was observed in the TFA treatment, but this occurred in a similar way in both soil regions, which would delay the leaching process of this element (Fig. 4).

With the uncontrolled use of RS in the production of crops, the emergence of soils with sodification problems becomes common, as has been reported in some areas of Pakistan that received RS for long periods, reaching contents of 1,525 mg dm^−3^ of Na^55^.

## Conclusion

The inversion in the position of application of raw sewage in the furrows (treatment with raw sewage applied with flow direction alternation in the furrow—TFA) provided a better distribution of nutrients in the soil along its length, which was confirmed by the fact that the nutrient and sodium contents in the soil did not show significant differences along the length of the furrows in the period of application of this wastewater.

In the conventional form of raw sewage application in the furrows, the application only at the beginning of the furrows (TFN) provided an increase in the TN and Na content in the soil at the inlet region, and the P and K contents showed an increase at the end of the furrows, raising the risk of leaching in these regions.

It is necessary to understand and raise awareness about the use of RS in agriculture by farmers, to reduce the risk of soil and groundwater contamination. More studies are recommended for this type of system to understand the behavior of toxic metals and microbiological contamination, among other pollutants.

## Supplementary Information

Supplementary Table 1.

Supplementary Table 2.

Supplementary Table 3.

## Data Availability

All data generated or analyzed during this study are included in this published article in the form of figures, tables and graphs.
