# Supplementary material for: Macronutrient distribution in soil subjected to raw sanitary sewage application by closed-end furrows
Source: Sci Rep. 2023 Jul 13;13:11323. doi: 10.1038/s41598-023-38528-5 (PMC10345096; doi:10.1038/s41598-023-38528-5)
Supplement: Supplementary file 1 — Supplementary Table 1. [file 41598_2023_38528_MOESM1_ESM.pdf]

Supllementary Material Table 1 - Data used to create the graphs in figure 2 of this work.

			Initial - 1st year			End - 1st year			Initial - 2nd year			End - 2nd year			Initial - 3rd year			End - 3rd year		
		Depths	Average	SD	Tukey	Average	SD	Tukey	Average	SD	Tukey	Average	SD	Tukey	Average	SD	Tukey	Average	SD	Tukey
TN (mg kg-1)	TWN	0 - 0.2 m	226.48	45.87	aB	219.62	61.11	aB	310.08	93.80	aA	268.97	59.82	aA	266.67	28.87	aB	250.02	50.01	aA
	TWA	0 - 0.2 m	212.10	11.24	aB	193.60	35.58	aB	300.26	70.47	aA	287.52	171.67	aAB	270.03	36.06	aB	260.00	50.24	aA
	TFN	0 - 0.2 m	346.88	56.49	aA	166.83	28.74	bB	366.73	25.95	aA	171.49	35.62	bB	420.05	34.64	bA	154.82	26.01	aB
	TFA	0 - 0.2 m	427.42	138.15	aA	451.45	148.88	aA	319.23	29.57	aA	327.07	109.17	aA	313.64	23.62	aB	286.22	23.87	aA

			Initial - 1st year			End - 1st year			Initial - 2nd year			End - 2nd year			Initial - 3rd year			End - 3rd year		
		Depths	Average	SD	Tukey	Average	SD	Tukey	Average	SD	Tukey	Average	SD	Tukey	Average	SD	Tukey	Average	SD	Tukey
P (mg kg-1)	TWN	0 - 0.2 m	14.66	4.20	aA	18.06	6.32	aA	12.75	3.84	aA	12.07	6.05	aB	15.49	3.84	aA	19.08	3.71	aA
	TWA	0 - 0.2 m	18.59	8.02	aA	14.79	10.01	aA	8.61	3.71	aA	11.79	1.77	aB	13.27	4.35	aA	13.99	6.00	aAB
	TFN	0 - 0.2 m	12.17	3.60	bA	26.06	3.28	aA	10.25	3.16	aA	24.89	5.00	bA	10.25	1.20	bA	26.46	6.70	aA
	TFA	0 - 0.2 m	16.36	5.72	aA	17.27	8.01	aA	16.59	3.11	aA	15.99	6.95	aAB	9.73	3.74	aA	10.94	0.91	aB

			Initial - 1st year			End - 1st year			Initial - 2nd year			End - 2nd year			Initial - 3rd year			End - 3rd year		
		Depths	Average	SD	Tukey	Average	SD	Tukey	Average	SD	Tukey	Average	SD	Tukey	Average	SD	Tukey	Average	SD	Tukey
K (mg kg-1)	TWN	0 - 0.2 m	39.33	4.84	aA	50.33	7.42	aA	78.87	9.64	aA	83.33	10.67	aA	73.33	25.17	aA	73.33	11.55	aA
	TWA	0 - 0.2 m	37.22	7.58	aA	43.44	6.34	aA	64.43	7.68	aAB	62.22	1.91	aB	63.33	23.09	aA	76.67	23.09	aA
	TFN	0 - 0.2 m	33.56	3.83	aA	43.67	6.06	aA	47.77	5.08	aC	65.47	20.11	aAB	23.33	15.28	bB	70.33	10.42	aA
	TFA	0 - 0.2 m	43.33	5.77	aA	46.67	4.84	aA	57.77	9.58	aBC	58.57	1.69	aB	60.22	36.06	aAB	53.33	25.17	aA

			Initial - 1st year			End - 1st year			Initial - 2nd year			End - 2nd year			Initial - 3rd year			End - 3rd year		
		Depths	Average	SD	Tukey	Average	SD	Tukey	Average	SD	Tukey	Average	SD	Tukey	Average	SD	Tukey	Average	SD	Tukey
Na (mg kg-1)	TWN	0 - 0.2 m	12.22	2.78	aB	13.67	4.16	aA	52.23	10.72	aB	72.23	12.64	aAB	50.23	45.83	aB	63.33	30.55	aA
	TWA	0 - 0.2 m	11.67	0.67	aB	13.67	3.79	aA	49.97	5.77	aB	51.23	19.51	aB	53.33	15.28	aB	33.33	23.09	aA
	TFN	0 - 0.2 m	25.11	7.18	aA	21.89	5.67	aA	160.67	40.13	aA	76.67	6.65	bAB	163.33	56.86	aA	53.33	11.55	bA
	TFA	0 - 0.2 m	23.67	5.29	aA	22.11	3.66	aA	100.67	27.27	aA	106.67	20.82	aA	136.67	32.15	aA	146.67	40.41	aB
