# Supplementary material for: Macronutrient distribution in soil subjected to raw sanitary sewage application by closed-end furrows
Source: Sci Rep. 2023 Jul 13;13:11323. doi: 10.1038/s41598-023-38528-5 (PMC10345096; doi:10.1038/s41598-023-38528-5)
Supplement: Supplementary file 2 — Supplementary Table 2. [file 41598_2023_38528_MOESM2_ESM.pdf]

Supllementary Material Table 2 - Data used to create the graphs in figure 3 of this work.

			Initial - 3rd year			End - 3rd year		
		**Depths**	**Average**	**SD**	**Tukey**	**Average**	**SD**	**Tukey**
pH	TWN	0 - 0.2 m	6.71	0.46	aA	6.75	0.20	aA
	TWA	0 - 0.2 m	6.31	0.04	aA	6.17	0.41	aA
	TFN	0 - 0.2 m	6.71	0.37	aA	6.76	0.53	aA
	TFA	0 - 0.2 m	6.74	0.45	aA	6.74	0.12	aA
			**Initial - 3rd year**			**End - 3rd year**		
		**Depths**	**Average**	**SD**	**Tukey**	**Average**	**SD**	**Tukey**
EC (µS cm-1)	TWN	0 - 0.2 m	47.85	3.57	aB	45.00	6.05	aA
	TWA	0 - 0.2 m	44.02	3.78	aB	43.00	7.09	aA
	TFN	0 - 0.2 m	70.01	15.34	aA	30.00	5.57	bB
	TFA	0 - 0.2 m	45.83	8.10	aB	49.75	5.54	aA
			**Initial - 3rd year**			**End - 3rd year**		
		**Depths**	**Average**	**SD**	**Tukey**	**Average**	**SD**	**Tukey**
OM (dag kg-1)	TWN	0 - 0.2 m	1.20	0.05	aB	1.30	0.08	aB
	TWA	0 - 0.2 m	1.20	0.10	aB	1.27	0.06	aB
	TFN	0 - 0.2 m	1.80	0.07	aA	1.30	0.05	bB
	TFA	0 - 0.2 m	1.70	0.09	aA	1.80	0.03	aA
