# Supplementary material for: Macronutrient distribution in soil subjected to raw sanitary sewage application by closed-end furrows
Source: Sci Rep. 2023 Jul 13;13:11323. doi: 10.1038/s41598-023-38528-5 (PMC10345096; doi:10.1038/s41598-023-38528-5)
Supplement: Supplementary file 3 — Supplementary Table 3. [file 41598_2023_38528_MOESM3_ESM.pdf]

Supllementary Material Table 3 - Data used to create the graphs in figure 4 of this work.

	treatments	Depths	Initial - 3rd year			End - 3rd year		
			Average	SD	Tukey	Average	SD	Tukey
TN (mg kg-1)	TWN	0 - 0.2 m	266.67	28.87	a	250.22	50.22	a
		0.2 - 0.4 m	228.67	114.22	a	298.59	202.09	a
		0.4 - 0.6 m	243.53	80.64	a	309.22	154.21	a
	TWA	0 - 0.2 m	270.76	36.06	a	260.84	50.53	a
		0.2 - 0.4 m	252.04	124.56	a	196.37	49.38	a
		0.4 - 0.6 m	311.40	84.41	a	275.37	94.33	a
	TFN	0 - 0.2 m	420.38	34.64	a	154.82	26.01	b
		0.2 - 0.4 m	328.12	76.17	b	193.69	92.46	ab
		0.4 - 0.6 m	295.35	138.14	b	241.38	31.04	a
	TFA	0 - 0.2 m	313.64	23.62	a	286.22	23.87	a
		0.2 - 0.4 m	292.65	83.24	a	268.10	25.70	a
		0.4 - 0.6 m	287.03	37.21	a	228.76	91.05	a
P (mg kg-1)	TWN	0 - 0.2 m	15.49	3.84	b	19.08	3.71	b
		0.2 - 0.4 m	27.88	6.53	a	24.38	4.66	a
		0.4 - 0.6 m	28.80	3.96	b	20.46	1.43	b
	TWA	0 - 0.2 m	13.27	4.35	b	13.99	6.00	b
		0.2 - 0.4 m	26.73	6.31	a	13.25	7.33	a
		0.4 - 0.6 m	15.89	6.24	b	17.53	3.69	b
	TFN	0 - 0.2 m	10.25	1.20	b	26.46	6.70	a
		0.2 - 0.4 m	18.82	4.01	a	19.81	5.02	ab
		0.4 - 0.6 m	21.03	6.43	a	17.53	2.55	b
	TFA	0 - 0.2 m	9.73	3.74	a	10.94	0.91	a
		0.2 - 0.4 m	12.04	3.73	a	17.60	8.57	a
		0.4 - 0.6 m	13.18	4.96	a	21.10	8.27	a
K (mg kg-1)	TWN	0 - 0.2 m	73.33	25.17	a	73.33	11.55	a
		0.2 - 0.4 m	116.67	35.12	a	60.33	26.46	a
		0.4 - 0.6 m	83.33	41.63	a	86.67	37.86	a
	TWA	0 - 0.2 m	63.33	23.09	a	76.67	23.09	a
		0.2 - 0.4 m	66.67	30.55	a	80.67	10.02	a
		0.4 - 0.6 m	66.67	20.82	a	50.33	17.32	a
	TFN	0 - 0.2 m	23.33	15.28	b	70.67	10.19	a
		0.2 - 0.4 m	76.67	20.82	a	70.67	10.19	a
		0.4 - 0.6 m	90.33	36.06	a	83.33	41.63	a
	TFA	0 - 0.2 m	60.33	36.06	a	53.33	25.17	a
		0.2 - 0.4 m	50.46	10.43	a	56.67	15.28	a
		0.4 - 0.6 m	60.33	20.41	a	46.67	35.12	a
Na (mg kg-1)	TWN	0 - 0.2 m	50.11	45.83	a	63.33	30.55	a
		0.2 - 0.4 m	46.67	15.28	a	20.11	10.84	a
		0.4 - 0.6 m	43.33	20.82	a	43.33	11.55	a
	TWA	0 - 0.2 m	53.33	15.28	a	33.33	23.09	a
		0.2 - 0.4 m	36.67	15.28	a	40.11	35.82	a
		0.4 - 0.6 m	66.67	30.55	a	40.11	17.32	a
	TFN	0 - 0.2 m	163.33	56.86	a	53.33	11.55	a
		0.2 - 0.4 m	173.33	5.77	a	76.67	11.55	a
		0.4 - 0.6 m	153.33	25.17	a	33.33	15.28	a
	TFA	0 - 0.2 m	136.67	32.15	a	146.67	40.41	a
		0.2 - 0.4 m	76.67	32.15	a	83.33	32.15	a
		0.4 - 0.6 m	110.02	17.32	a	100.74	10.84	a
